# Supplementary material for: Characterization of Zr-Containing Dispersoids in Al–Zn–Mg–Cu Alloys by Small-Angle Scattering
Source: Materials (Basel). 2023 Jan 31;16(3):1213. doi: 10.3390/ma16031213 (PMC9919802; doi:10.3390/ma16031213)
Supplement: Supplementary file 1 [file materials-16-01213-s001.zip › materials-2125429-supplementary final.pdf]

Supplementary Information

Characterization of Zr-Containing Dispersoids in Al-Zn-Mg-Cu Alloys by Small-Angle Scattering

Mohammad Taha Honaramooz[1], Roland Morak[2,*], Stefan Pogatscher[3], Gerhard Fritz-Popovski[1], Thomas Kremmer[3], Thomas Meisel[4], Johannes A. Österreicher[5], Aurel Arnoldt[5], Oskar Paris[1,*]

1 Chair of Physics, Department Physics, Mechanics and Electrical Engineering, Montanuniversität Leoben, Franz-Josef-Str. 18, 8700 Leoben, Austria

2 R&D Material Science CMI, Center for Material Innovation, AMAG Rolling GmbH, Postfach 32, 5282 Ranshofen, Austria

3 Chair of Nonferrous Metallurgy, Department Metallurgy, Montanuniversität Leoben, Franz-Josef-Str. 18, 8700 Leoben, Austria

4 Chair of General and Analytical Chemistry, Department General, Analytical and Physical Chemistry, Montanuniversität Leoben, Franz-Josef-Str. 18, 8700 Leoben, Austria

5 LKR Light Metals Technologies, Austrian Institute of Technology, Lamprechtshausenerstraße 61, 5282 Ranshofen, Austria

*Corresponding authors

S1- SAXS and SANS profiles

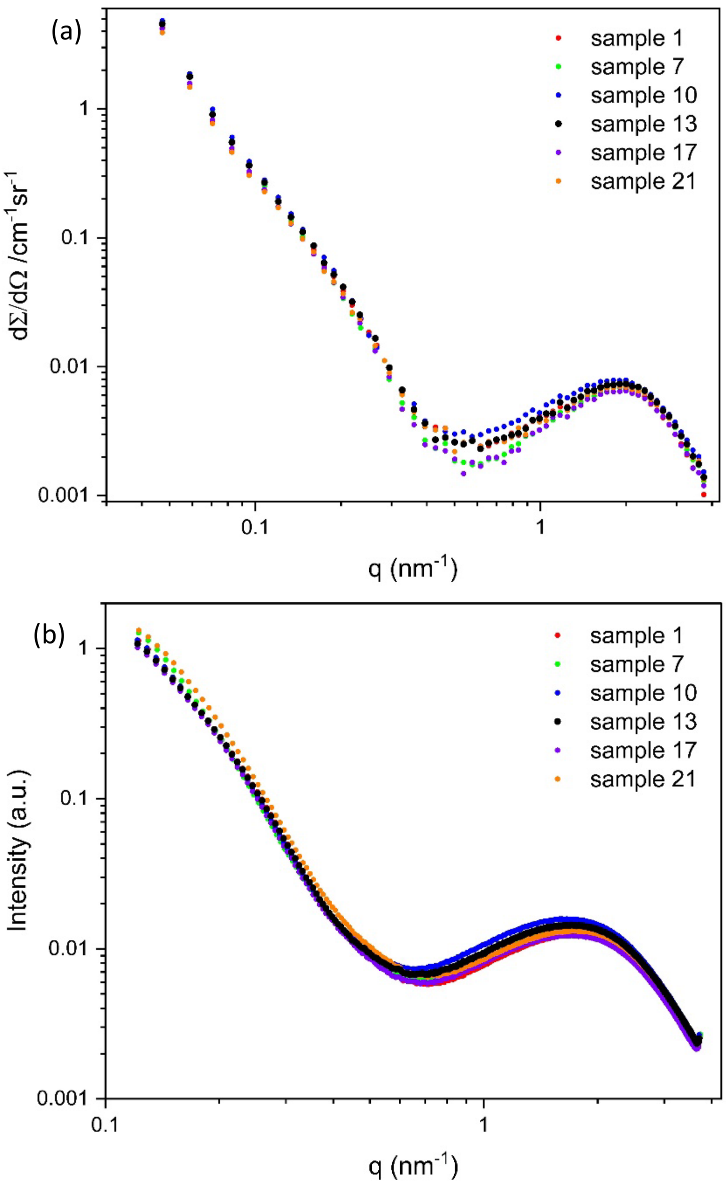

Figure S1-(a) SANS curves and (b) SAXS curves from selected samples

S2- TEM images

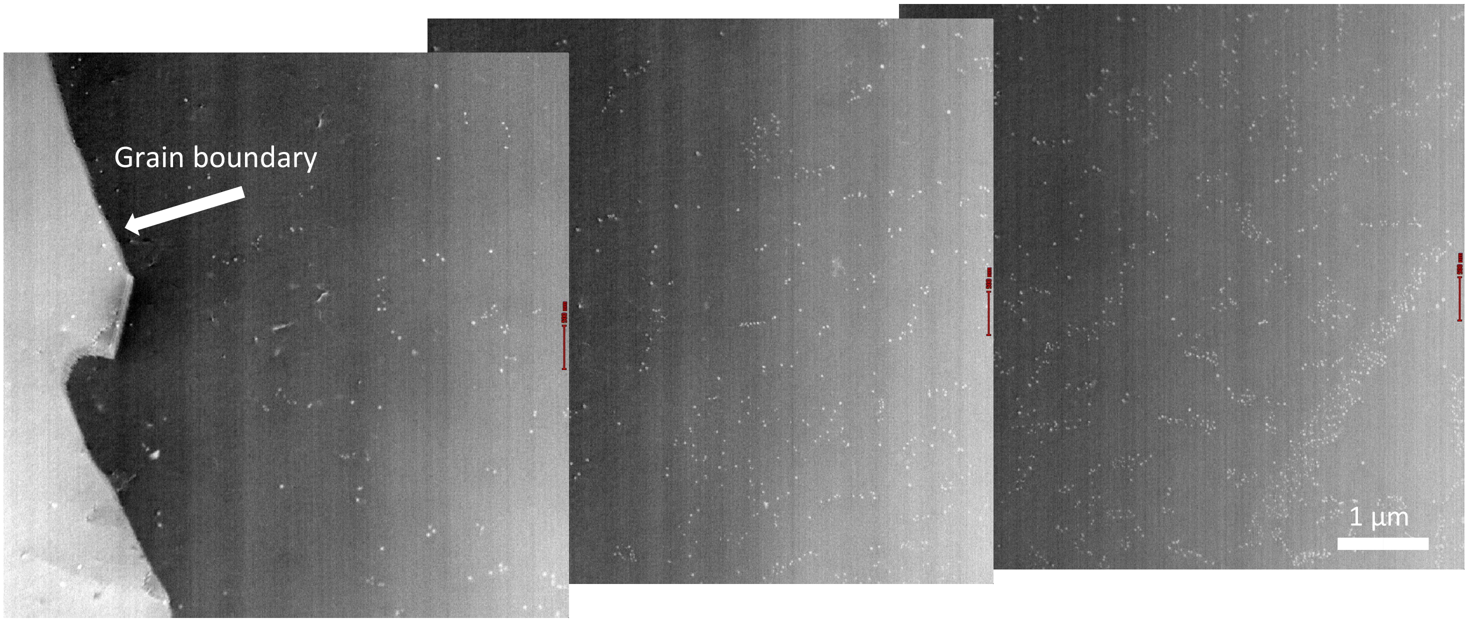

Figure S2- HAADF-STEM images of sample No. 10 in dark field mode, showing the distribution of Zr-containing dispersoids from a grain boundary towards the middle of the grain

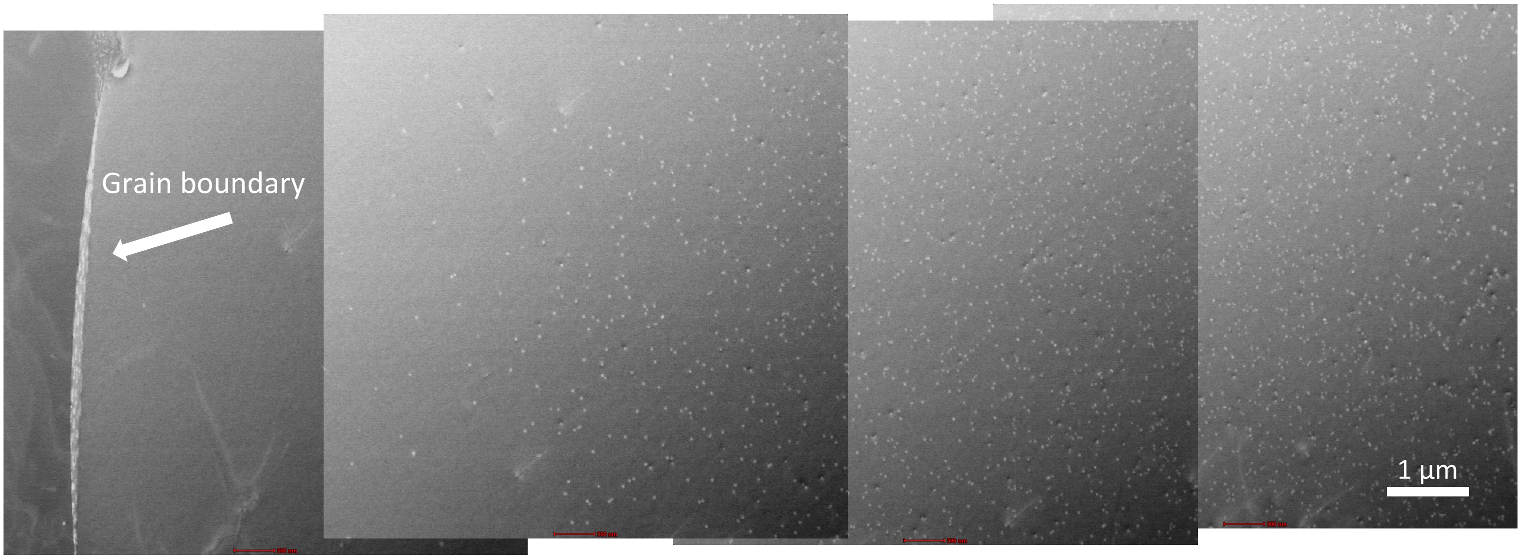

Figure S3- HAADF-STEM images of sample No. 1 in dark field mode, showing the distribution of Zr-containing dispersoids from a grain boundary towards the middle of the grain.

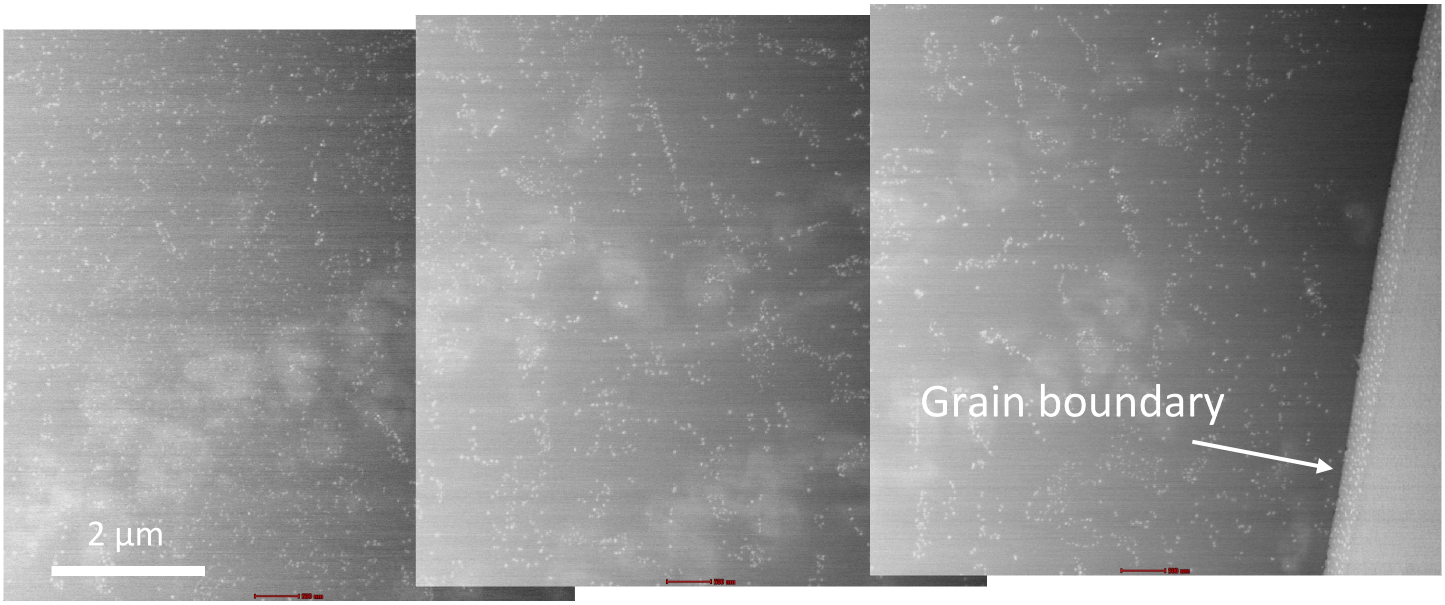

Figure S4-- HAADF-STEM images of sample No. 13 in dark field mode, showing the distribution of Zr-containing dispersoids from a grain boundary towards the middle of the grain

S3 Mathematical Background of SAS Data Evaluation

The small angle scattering data from the samples shown in Figure S1 are described by the sum of four contributions

$$I_{fit}(q) = I_S(q) + I_D(q) + I_{GP}(q) + k_B \tag{S1}$$

where $I_S(q)$ is the contribution from the S-phase or other large primary precipitates, $I_D(q)$ the contribution from the dispersoids, $I_{GP}(q)$ the one due to the GP zones, and k_B a constant background.

The S-phase precipitates are large (>100 nm), which leads to $I(q) = k_S \cdot q^{-4}$ within the measured q-range [1], with the unknown proportionality constant k_S.

The contribution by the GP zones is modelled according to Fratzl et al. [2-3] as

$$I_{GP}(q) = k_{GP} \cdot \frac{a\left(\frac{q}{q_{GP}}\right)^4}{\left(\frac{q}{q_{GP}}\right)^4 + c} \cdot \frac{b}{b + \left(\left(\frac{q}{q_{GP}}\right)^2 - 1 + d\right)^2} = k_{GP} \cdot G(q) \tag{S2}$$

with

$$a = (1+c)\left(1 + \frac{d^2}{b}\right); \quad b = \left(\frac{2\gamma(1-d)}{1-\gamma^2}\right)^2; \quad c = \frac{d}{b - d(1-d)}; \tag{S3}$$

$$\gamma = \frac{\sigma}{8\pi\varphi(1-\varphi_{GP})}; \quad \sigma = \begin{cases} 4\pi\left(\frac{\varphi_{GP}}{\pi}\right)^{2/3} & \varphi_{GP} < \frac{4\pi}{81} \\ 2\pi\sqrt{\frac{\varphi_{GP}}{\pi}} & \frac{4\pi}{81} \leq \varphi_{GP} \leq \frac{1}{\pi} \\ 2 & \varphi_{GP} > \frac{1}{\pi} \end{cases}$$

Since several of these parameters depend on each other, $I_{GP}(q)$ can be uniquely expressed by the pre-factor k_{GP}, the volume fraction of GP-zones φ_{GP}, and the position of the maximum q_{GP}, where the parameter d was set to $d = 0.06$ [4].

Finally, $I_D(q)$ is modeled using a homogeneous distribution of non-interacting spherical precipitates with a free-form radius-distribution $D(r)$. The size distribution $D(r)$ of the dispersoids can be represented as the sum of n basis functions $\chi_i(r)$ using the prefactors f_i

$$D(r) = \sum_{i=1}^{n} f_i \cdot \chi_i(r) \tag{S4}$$

We used cubic B-slines as basis functions. If these basis functions are transformed in reciprocal space by using the form factor of spheres

$$\psi_i(q) = \int_0^\infty \chi_i(r) \cdot r^w \cdot \left(3\frac{\sin(qr) - qr\cos(qr)}{q^3 r^3}\right)^2 dr \tag{S5}$$

one can represent $I_D(q)$ as

$$I_D(q) = \sum_{i=1}^{n} f_i \cdot \psi_i(q) \tag{S6}$$

where the exponent w can be used to obtain the distribution weighted by number ($w = 6$), volume ($w = 3$) or intensity ($w = 0$) [5].

The coefficients k_S, k_{GP}, k_B, f_1 ... f_n from Eq. S1 are obtained by solving the equation system similar to the Indirect Fourier transformation technique

$$(\mathbf{B} + \lambda\mathbf{S})\mathbf{f} = \mathbf{d} \tag{S7}$$

where the **c** is a vector containing the solution coefficients

$$\mathbf{f} = \begin{pmatrix} f_1 \\ \vdots \\ f_n \\ k_S \\ k_G \\ k_B \end{pmatrix} \tag{S8}$$

and where

$$\mathbf{B} = \mathbf{A^T G A} \qquad \mathbf{d} = \mathbf{A^T G I} \tag{S9}$$

with

$$\mathbf{I} = \begin{pmatrix} I(q_1) \\ I(q_2) \\ \vdots \\ I(q_m) \end{pmatrix} \qquad \mathbf{A} = \begin{pmatrix} \psi_1(q_1) & \psi_2(q_1) & \cdots & \psi_n(q_1) & 1/q_1^4 & G(q_1) & 1 \\ \psi_1(q_2) & \psi_2(q_2) & \cdots & \psi_n(q_2) & 1/q_2^4 & G(q_n) & 1 \\ \vdots & \vdots & \ddots & \vdots & \vdots & \vdots & \vdots \\ \psi_1(q_m) & \psi_2(q_m) & \cdots & \psi_n(q_m) & 1/q_m^4 & G(q_m) & 1 \end{pmatrix} \tag{S10}$$

$$\mathbf{G} = \begin{pmatrix} \frac{1}{\sigma_1^2} & 0 & \cdots & 0 \\ 0 & \frac{1}{\sigma_2^2} & \cdots & 0 \\ \vdots & \vdots & \ddots & \vdots \\ 0 & 0 & 0 & \frac{1}{\sigma_m^2} \end{pmatrix} \qquad \mathbf{S} = \begin{pmatrix} 1 & -1 & 0 & \cdots & 0 & 0 & 0 & 0 & 0 \\ -1 & 2 & -1 & \cdots & 0 & 0 & 0 & 0 & 0 \\ 0 & -1 & 2 & \cdots & 0 & 0 & 0 & 0 & 0 \\ \vdots & \vdots & \vdots & \ddots & \vdots & \vdots & \vdots & \vdots & \vdots \\ 0 & 0 & 0 & \cdots & 2 & -1 & 0 & 0 & 0 \\ 0 & 0 & 0 & \cdots & -1 & 1 & 0 & 0 & 0 \\ 0 & 0 & 0 & \cdots & 0 & 0 & 1 & 0 & 0 \\ 0 & 0 & 0 & \cdots & 0 & 0 & 0 & 1 & 0 \\ 0 & 0 & 0 & \cdots & 0 & 0 & 0 & 0 & 1 \end{pmatrix}$$

using the measured intensities $I(q_j)$ with their limit of uncertainty σ_j at the m points at q_j. The parameter λ is a Lagrange multiplier, which determines the stabilization of the size distribution. This stabilization is done by matrix **S**, which links the values of the prefactor of the basis function f_i to the prefactors of the neighbouring basis functions f_{i-1} and f_{i+1}, but does not impose such a constraint of the coefficients k_S, k_G, and k_B.

A whole range of different Lagrange multipliers is used with λ values changing over several orders of magnitude. For each λ equation (S7) is solved repeatedly with varying parameters q_{GP} and φ_{GP} until the lowest mean deviation M has been reached with

$$M = \sqrt{\frac{1}{m}\sum_{j=1}^{m}\frac{\left(I_{fit}(q_j) - I(q_j)\right)^2}{\sigma_j^2}} \tag{S11}$$

Since the approximation of the parameters q_{GP} and φ_{GP} corresponds to a non-linear least squares problem, the Boltzmann simplex simulated annealing algorithm to find the best solution [6].

The correct Lagrange multiplier is selected by searching a solution that combines a low mean deviation with $\frac{dN_c}{d\lambda} \approx 0$, where

$$N_c = \sum_{i=1}^{n-1}|c_{i+1} - c_i| \tag{S12}$$

S4 Comparison of SAXS and SANS

Generally, the SAS intensity of a two-phase system of precipitates P in a matrix M can be written as [7]

$$I(q) = \varphi_P\varphi_M(\rho_M - \rho_P)^2F(q) = \varphi_P(1 - \varphi_P)KF(q) \tag{S13}$$

Where φ_P and φ_M are the volume fractions, $K = (\Delta\rho)^2 = (\rho_M - \rho_P)^2$ is the scattering contrast between matrix and precipitates, $\Delta\rho$ being the difference of their scattering length densities, and $F(q)$ is the square of the scattering amplitude. We use the following approximations for the scattering of two independent types of precipitates, i.e. dispersoids (D) and GP-zones (GP) in an Al-rich matrix:

a) The two precipitate phases do not interact with each other such that their scattering intensities can be added (incoherent approximation).

b) The two precipitate phases are dilute, which results in $\varphi_M = (1 - \varphi_P) \approx 1$ with the scattering length density of the matrix being approximately the one of pure Al, $\rho_M \approx \rho_{Al}$, for both. This means that we can employ two independent 2-phase approximations for D and GP.

c) The two precipitate phases have a considerably different size, such that their intensity distributions are well separated in reciprocal space.

Using approximations a) and b) we can write for the scattering intensity of dispersoids and GP-zones measured with SANS (upper index *n*) and SAXS (upper index *x*), respectively

$$I^n(q) = C^n\left(\varphi_D K_D^n F_D(q) + \varphi_{GP} K_{GP}^n F_{GP}(q)\right) \tag{S14}$$

$$I^x(q) = C^x\left(\varphi_D K_D^x F_D(q) + \varphi_{GP} K_{GP}^x F_{GP}(q)\right) \tag{S15}$$

With C^n and C^x being experimental constants and the lower indices *D* and *GP* refer to dispersoids and GP-zones, respectively. For the ratio of the intensities we obtain with $C = \frac{C^n}{C^x}\frac{K_D^n}{K_D^x}$

$$\frac{I^n(q)}{I^x(q)} = C\frac{\left(F_D(q)+\frac{\varphi_{GP}}{\varphi_D}\cdot\frac{K_{GP}^n}{K_D^n}\cdot F_{GP}(q)\right)}{\left(F_D(q)+\frac{\varphi_{GP}}{\varphi_D}\cdot\frac{K_{GP}^x}{K_D^x}\cdot F_{GP}(q)\right)} \quad \text{(S16)}$$

This expression generally depends on both, the *contrast ratios* and *the ratio of the volume fractions* of the two precipitate types. However, when considering that the dispersoids are much larger than the GP-zones (approximation c)), their scattering can be neglected in the large q-region where the scattering from GP-zones dominates. Therefore

$$\frac{I^n(q)}{I^x(q)} \approx C\frac{K_{GP}^n}{K_D^n}\frac{K_D^x}{K_{GP}^x} \quad \text{(S17)}$$

for large values of q, were the scattering from GP-zones is dominant. Please note that this expression is now independent of the volume fractions and depends on the contrast ratios only. Table S1 summarizes the estimates of the scattering contrasts for GP-zones and dispersoids, leading to $\frac{I^n}{I^x} \approx 4.5$ in Eq. S17, in good agreement with the experimental result shown in Figure 2 in the main manuscript. For comparison, Table S1 lists also the contrast scenario for the η-phase. In this case one would expect a factor 1.7 for I^n/I^x in Eq. S17. Therefore, it is concluded that the scattering in the region of the first hump at intermediate q in Figure 2 and Fig. 3a,b in the main manuscript stems dominantly from Zr-rich dispersoids, allowing to evaluate the SAS curves quantitatively towards the size distribution of dispersoids as described in S3.

Table S1 - Scattering length densities ρ for X-rays (index x) and neutrons (index n) (from Ref. [8]), and scattering contrast $K = (\Delta\rho)^2$ of precipitate phases with respect to pure aluminum.

Phase	Composition [9] (approximated)	ρ_x (10^{-6}/Å^2)	ρ_n (10^{-6}/Å^2)	K_x (10^{-12}/Å^4)	K_n (10^{-12}/Å^4)
Matrix	Al	22.44	2.08	-	-
GP-Zones	$Al_{25}Zn_{36}Mg_{34}Cu_5$ [10]	32.28	3.08	97	1
Dispersoids	Al_3Zr	32.66	2.55	104	0.22
η-Phase	$MgZn_2$	38.46	3.31	256	1.5

S5 Supplementary References

[1] G. Porod, "Die Röntgenkleinwinkelstreuung von dichtgepackten kolloiden Systemen - I. Teil," *Kolloid-Zeitschrift*, vol. 124, no. 2, pp. 83–114, 1951, doi: 10.1007/BF01512792.

[2] P. Fratzl, J. L. Lebowitz, O. Penrose, and J. Amar, "Scaling functions, self-similarity, and the morphology of phase-separating systems," *Phys. Rev. B*, vol. 44, no. 10, pp. 4794–4811, 1991, doi: 10.1103/PhysRevB.44.4794.

[3] P. Fratzl, "J. Appl. Cryst. (1991). 24, 593-597 Volume-Fraction Dependence of the Scaling Function for Phase-Separating Systems," pp. 593–597, 1991.

[4] P. Fratzl and J. L. Lebowitz, "Universality of scaled structure functions in quenched systems undergoing phase separation," *Acta Metall.*, vol. 37, no. 12, pp. 3245–3248, 1989, doi: 10.1016/0001-6160(89)90196-X.

[5] Glatter, O., "Computation of Distance Distribution Functions and Scattering Functions of Models for Small Angle Scattering Experiments", *Acta Phys. Aust.*, 1980, **52**, 243-256.

[6] Press, W. H.; Teukolsky, S. A.; Vetterling, W. T. & Flannery, B. P. Numerical Recipes in Fortran 77, Cambridge University Press, 1986

[7] O. Glatter and O. Kratky (Ed.), Small-Angle X-ray Scattering, London, Academic Press, 1982.

[8] https://www.ncnr.nist.gov/resources/activation/

[9] https://materials.springer.com/

[10] P. Schloth, J. N. Wagner, J. L. Fife, A. Menzel, J. M. Drezet, and H. Van Swygenhoven, "Early precipitation during cooling of an Al-Zn-Mg-Cu alloy revealed by in situ small angle X-ray scattering," *Appl. Phys. Lett.*, 2014, 105, 2–5.
